# Breastfeeding and Human Lactation

**DOI:** 10.3390/nu11040802

**Published:** 2019-04-09

**Authors:** Donna Geddes, Sharon Perrella

**Affiliations:** School of Molecular Sciences, M310, The University of Western Australia, Crawley, Perth, WA 6009, Australia; sharon.perrella@uwa.edu.au

**Keywords:** Breastfeeding, human lactation, lactation, human milk, breast milk, milk composition

## 1. Introduction

Breastfeeding is the very means by which humans have thrived and developed as a species. Indeed, the Developmental Origins of Health and Disease Hypothesis recognises that the breastfeeding phase, which can continue to 2 years and beyond, plays a major role in the continuum of optimal programming of the lifelong health and development of the infant. Early life nutrition therefore presents a window of opportunity where the infant’s short and long-term health can potentially be improved in the face of escalating rates of chronic disease that have reached epidemic proportions.

This special issue “Breastfeeding and Human Lactation” is thus timely, in an era of resurgence of lactation research, and is comprised of 30 manuscripts that cover a wide range of areas. This research will contribute to a growing scientific knowledge base that is critical to improving breastfeeding rates and the delivery of human milk (HM) to all infants, including those that cannot breastfeed, such as the vulnerable preterm infant. The majority of the papers in this issue address one of two broad themes; factors influencing milk composition, or relationships between milk composition and infant development. Findings from these research papers further elucidate the variability of milk composition and its impact on infant health.

## 2. Factors Influencing Milk Composition

It is evident that mammalian milk evolved as a protective fluid harbouring antimicrobial proteins predominantly for the protection of the offspring, with nutrition developing later. As such, many components of milk have dual roles, working synergistically to protect and nourish the infant. Indeed, the footprints of evolution are apparent in the presence of immune cells in HM that increase significantly in response to both maternal and infant infections. Twigger et al. [1] have identified antimicrobial proteins, granulysin and perforin along with other granzymes released by leukocytes in HM, that are elevated in maternal breast infection. Milk immune cells may therefore be beneficial for protection of both the infant and the breast. 

Anti-secretory factor (AF) is involved in the regulation of secretory processes and inflammation and is expressed in immune cells: B-cells, macrophages and dendritic cells. AF concentrations in HM are lower than that of maternal plasma, with a positive relationship between milk AF concentration and maternal body mass index (BMI), which might be due to a greater level of maternal inflammation associated with obesity [2]. 

It is increasingly apparent that maternal factors such as body composition, diet, ethnicity, geography, genetics and lifestyle all contribute to the unique milk signature of each woman. In this issue, a number of papers have shown differences in milk composition with respect to geographical location. In particular, concentrations of the immune active molecules transforming growth factor-β2 (TGF-β2), immunoglobulin A (IgA), and hepatocyte growth factor (HGF) were higher in African women than in Italian women, suggesting a stronger response to the environment and thus greater infant protection against infection [3]. 

With cutting-edge technologies, it is possible to study metabolites in all bodily fluids. Variability of the metabolite profile of HM has not been comprehensively explored, however three papers have attempted this ambitious task. It was shown in two studies that the milk metabolome differs according to country [4,5] and mode of birth. Further interactions between the milk metabolites and microbes in the milk were also discovered, indicating the importance of the milk microbiome [5]. The third paper studied the endocannabinoid metabolome, for which there are receptors in the infant brain with evidence of a role in appetite and food intake. The study aimed to determine differences in endocannabinoids between transitional and mature milk, of which only one was significant [6]. The impact of these components on infant growth and development is yet to be studied.

Variability in HM composition would logically depend on maternal diet to some extent, although few studies have been carried out in this area. Studies that attempted this difficult task have provided conflicting results, largely due to the observational nature of the research. Two papers in this issue demonstrated an absence of relationship between diet and macronutrients [7,8]. Similarly, no relationships were observed between maternal dietary intakes of the micronutrients choline and zinc and their respective HM concentrations [9]. For breastfeeding women in a population with a high prevalence of zinc deficiency, zinc supplementation during pregnancy did not impact postnatal maternal serum zinc levels, which likely reflect HM concentrations [10]. Relationships were noted between diet and HM fatty acid profiles, as previously documented [8]. Interestingly, Bzikowska-Jura et al made the observation that maternal adiposity was related to HM protein and energy content at 3 months lactation, irrespective of diet [11]. Appropriate HM sampling methods are imperative when examining variability of milk components. In this context, Bzikowska-Jura et al found a weak relationship between HM fat content and maternal BMI using an intense sampling regime to account for changes in fat over the course of 24 h. Kent et al trialed hourly expression of breast milk over 3 h (4 expressions) in an effort to estimate rates of milk fat synthesis. Unfortunately, this was not a reliable measure when compared to 24 h milk sampling [12]. George et al has highlighted sampling as one of the major challenges when examining milk lipids [13].

The idea of maternal-infant signaling via milk is attractive to explain both milk composition and infant outcome variability. Maternal adiposity is related to lower lean infant mass across 12 months of lactation [14], and while a review in the issue suggests that milk is tailored according to sex of the infant, there is yet to be strong evidence of this in humans [15].

## 3. Relationships of Milk Composition with Infant Protection, Growth and Development

Historically very few milk components have been associated with infant outcomes. Two papers in this issue highlight that the dose, rather than concentrations, of milk components are associated with infant body composition development over the first 12 months of life. Specifically, Gridneva and colleagues showed that the 24 h dose of appetite hormones adiponectin and whole milk leptin [16], along with casein [17], are differentially related to the development of infant body composition. The mechanisms by which the components exert their effects are still not clear. 

Interestingly, endogenous satiety factors produced in the small intestine have been detected in HM and have been related to infant weight gain and weight for age z scores [18]. Whilst more work has to be done to verify the results, it is becoming increasingly clear that both the composition and volume of milk consumed by the infant modulates growth and development.

Growth of the preterm infant is critical, as these vulnerable infants are at high risk for morbidities both early and later in life. Whilst HM is recommended as the optimal nutrition for preterm infants, fortification is almost universal to ensure adequate growth of those born < 33 weeks gestation. The delivery of human milk during continuous enteral feeding therefore is an area where enhancement may be needed to avoid the potential loss of nutrients to the infant. Zozaya et al. [19] found a reduction in the total fat delivered to the preterm infant via continuous enteral feeding, with long chain fatty acids more likely to be adsorbed to the feeding tube. These losses, while statistically significant, were considered clinically small. Once preterm infants are able to feed orally a dilemma exists about how to feed the infant in the absence of the breastfeeding mother. Geddes et al compared breastfeeding with use of a novel teat that required the infant to apply a vacuum and use a tongue movement mimicking that of breastfeeding to remove milk. They observed that although the infants’ intra-oral vacuums were lower with the teat than at the breast, more milk was transferred [20]. This finding is indicative of the immaturity of the preterm infant’s oral motor systems and should be taken into account when transitioning to full breastfeeding.

Many of the preterm infant’s systems are immature, in particular the gastrointestinal system. This increases the preterm infant’s susceptibility to infection and may impact the digestion of milk and subsequent absorption of nutrients and immune components. Indeed, Demers-Mathiew et al. [21] have described differences in the digestion of HM immunoglobulins between the preterm and term infant. The impacts of these findings are yet to be determined. 

One of the major reasons HM feeding is recommended for preterm infants is that it markedly decreases the risk for necrotizing enterocolitis. However, controversy exists over whether raw or pasteurized HM should be fed to infants less than 32 weeks corrected age or less than 1500g in weight due to the high prevalence of cytomegalovirus in the milk. Lopes et al. [22] describe the heterogeneity in feeding practices between French neonatal units highlighting lack of consensus within the medical field. Pasteurization of HM is of concern because it reduces the impact of several immune factors in milk, including lactoferrin, which plays a significant role in antimicrobial and immunomodulatory functions. Telang provided a comprehensive review of the structure and functions of lactoferrin, and discussed the importance of continued clinical trials in determining the role of lactoferrin in prevention of neonatal sepsis [23]. 

An in-depth understanding of both the complex processes that impact HM and the impact of HM on the infant is critical to understanding lactation dysfunction, and may inform the identification of windows of potential intervention. An understanding of physiological and clinical dilemmas in lactation is also important.

In this context, insufficient milk supply is the most common reason for early weaning. Currently evidence-based treatments are limited for women with low milk supply. While galactogogues are often prescribed, the effect is modest for pharmacologic galactogogues as reviewed by Asztalos [24]. In light of this review, much more research is required to understand the causes of low milk supply along with more controlled studies of the efficacy of galactogogues. 

Low milk supply may follow delayed secretory activation, or may be associated with breast inflammation. While both conditions are characterised by an elevated HM sodium concentration and sodium:potassium ratio, to date there are no clinical tools available to track these complications of lactation. Lai et al validation of handheld devices for determining sodium and potassium levels in HM indicates these may offer a promising point of care tool for monitoring secretory activation, the onset of mastitis and evaluation of treatment [25]. 

Mothers face many other barriers to successful breastfeeding, including their perceptions and own wellbeing [26,27]. Early hospital practices can also impact lactation, including early introduction of formula in the hospital, which was estimated at 28% in the UK [28]. The authors found many of the factors implicated in early supplementation to be modifiable. Further early recognition of infant feeding cues and responsive feeding is facilitated by increased mother-infant contact [29]. 

Finally, one must not discount the health benefits reaped by the lactating mother. The incidence of gestational diabetes mellitus is increasing and is associated with greater maternal risk for type 2 diabetes. However, breastfeeding is associated with lower risk of maternal type 2 diabetes, and in a new analysis maternal thyroid function also appears to be positively affected out to 6–16 years post-partum [30]. 

## 4. Conclusions

While HM is traditionally thought of primarily as a source of infant nutrition, evidence from lactation research shows a diverse range of functions, including protection from infection and disease, and programming of future health and development of both mother and infant through microbial and hormonal signaling. Interactions between maternal endocrine and mammary function, as well as diet, also impact milk composition and production. New evidence presented in this special edition of Nutrients contributes to the growing body of lactation and breastfeeding research, and informs our understanding of the complex composition of HM and its impact on infant health.
